# Crystal structure of *N* ^1^-phenyl-*N* ^4^-[(*E*)-(pyren-1-yl)methyl­idene]benzene-1,4-di­amine

**DOI:** 10.1107/S2056989015001814

**Published:** 2015-02-07

**Authors:** Md. Serajul Haque Faizi, Elena V. Prisyazhnaya

**Affiliations:** a Department of Chemistry, Indian Institute of Technology Kanpur, Kanpur, UP 208 016, India; b Department of Chemistry, Kyiv National University of Construction and Architecture, Povitroflotsky Avenue 31, 03680 Kiev, Ukraine

**Keywords:** crystal structure, *N*-phenyl-*p*-phenyl­enedi­amine, 1-pyrenecarboxaldehyde, PMBD, Schiff base, N—H⋯π and C—H⋯π inter­actions, π–π inter­actions

## Abstract

In the title compound, C_29_H_20_N_2_, the dihedral angles subtended by the central *p*-phenyl­enedi­amine ring with respect to the mean plane of the terminal pyrenyl ring system (r.m.s. deviation = 0.027 Å) and the terminal *N*-phenyl ring are 29.34 (4) and 43.43 (7)°, respectively. The conformation about the C=N bond is *E*. In the crystal, mol­ecules are linked by N—H⋯π and C—H⋯π inter­actions forming chains propagating along the [10-2] direction. These chains are linked *via* π–π inter­actions [inter-centroid distances are in the range 3.5569 (11)–3.708 (1) Å], forming slabs lying parallel to (30-4).

## Abstract 2

The title compound is non-planar, with the mean plane of the pyrene ring system and the terminal *N*-phenyl ring being inclined to the central *p*-phenyl­enedi­amine ring by 29.34 (4) and 43.43 (7)°, respectively. In the crystal, mol­ecules are linked by a number of weak N—H⋯π, C—H⋯π and π–π inter­actions [inter-centroid distances = 3.5569 (11)–3.708 (1) Å], forming slabs lying parallel to (30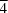).

## Chemical context   

Schiff bases often exhibit various biological activities, and in many cases have been shown to have anti­bacterial, anti­cancer, anti-inflammatory and anti­toxic properties (Lozier *et al.*, 1975 ▸). They are used as anion sensors (Dalapati *et al.*, 2011 ▸), as non-linear optical compounds (Sun *et al.*, 2012 ▸) and as versatile polynuclear ligands for multinuclear magnetic exchange clusters (Moroz *et al.*, 2012 ▸). The pyrene unit is one of the most commonly used fluoro­phores due to its strong luminescence and chemical stability (Aoki *et al.*, 1991 ▸; Nishizawa *et al.*, 1999 ▸; van der Veen *et al.*, 2000 ▸). Another inter­esting feature of the pyrene unit is the π–π inter­action between pyrene aromatic rings in the crystal packing, which can permit the formation of highly ordered mol­ecular aggregates in the solid state by architecturally controlled self-assembly (Desiraju *et al.*, 1989 ▸; Munakata *et al.*, 1994 ▸). Pyrene is a commonly used fluoro­phore due to its unusual fluorescent properties: intense fluorescence signals, vibronic band dependence with the media (Karpovich & Blanchard, 1995 ▸), and use in fluorescence sensors (Bell & Hext, 2004 ▸) and excimer formation (Lodeiro *et al.*, 2006 ▸). As a result of these particular properties and because of its chemical stability, it is also employed as a probe for solid-state studies (Corma *et al.*, 2002 ▸) and polymer association (Seixas de Melo *et al.*, 2003 ▸). We report herein on the crystal structure of the title compound, synthesized by the condensation reaction of 1-pyrenecarboxaldehyde and *N*-phenyl-*p*-phenyl­enedi­amine.
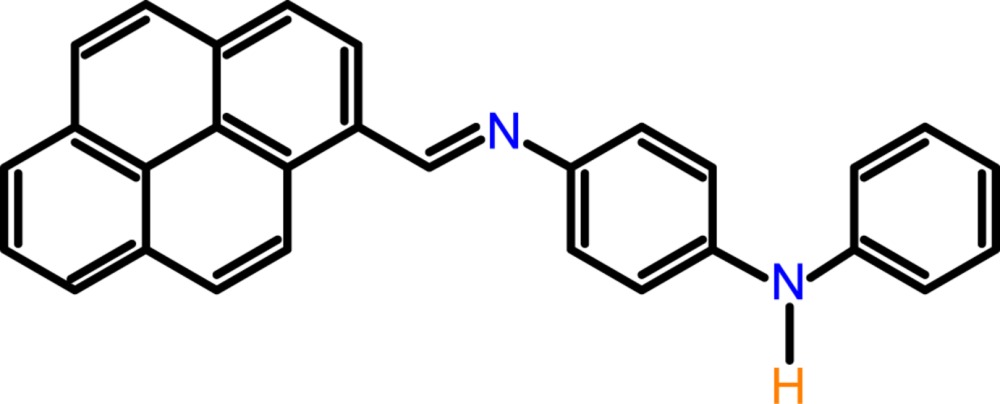


## Structural commentary   

**Figure 1:**
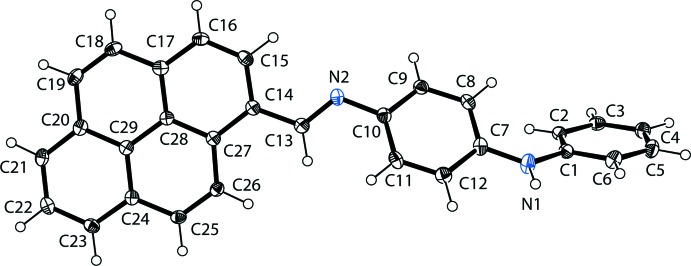
The mol­ecular structure of the title compound, showing the atom labelling. Displacement ellipsoids are drawn at the 40% probability level.

The mol­ecular structure of the title compound is illustrated in Fig. 1 ▸. The compound is non-planar, the dihedral angles between the central benzene ring (C7–C12) and the terminal phenyl ring (C1–C6) and the mean plane of the pyrenyl ring system (C14–C29; r.m.s. deviation = 0.027 Å) being 43.43 (9) and 29.33 (7)°, respectively. The conformation about the C13=N2 bond is *E* with a C10—N2—C13—C14 torsion angle of 178.13 (15)°.

## Supra­molecular features   

**Figure 2:**
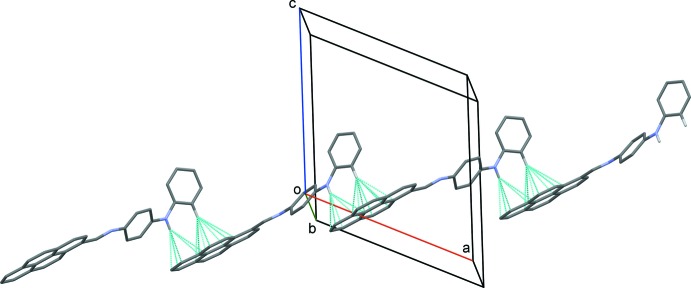
A view along the *b* axis of the zigzag chain in the crystal of the title compound. The C—H⋯π and N—H⋯π inter­actions are shown as dashed lines (see Table 1 ▸ for details).

**Figure 3:**
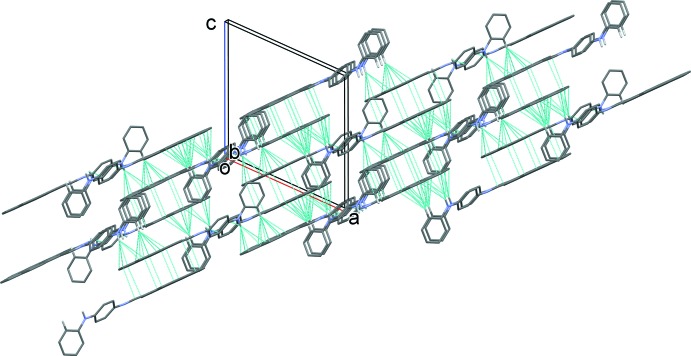
A view along the *b* axis of the crystal packing of the title compound. The C—H⋯π, N—H⋯π and π–π inter­actions are shown as dashed lines (see Table 1 ▸ for details).

In the crystal, mol­ecules are connected *via* N—H⋯π and C—H⋯π inter­actions forming zigzag chains propagating along [10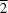]; see Table 1 ▸ and Fig. 2 ▸. These chains are linked *via* π–π inter­actions involving inversion-related pyrenyl rings, forming two-dimensional networks lying parallel to (30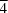); see Fig. 3 ▸. The inter-centroid distances are 3.7051 (11), 3.708 (1), 3.6905 (11) and 3.5569 (11) Å for π–π inter­actions involving *Cg*3⋯*Cg*5^ii^, *Cg*3⋯*Cg*6^ii^, *Cg*4⋯*Cg*6^ii^ and *Cg*6⋯*Cg*6^ii^, respectively, where *Cg*3, *Cg*4, *Cg*5 and *Cg*6 are the centroids of the C14–C17/C28–C27, C17–C20/C28–C29, C20–C24/C29 and C24–C29 rings, respectively [symmetry code: (ii) = −*x* + 1, −*y*, −*z*]. Inter­action *Cg*6⋯*Cg*6^ii^ is a slipped parallel π–π inter­action with an inter­planar distance of 3.3614 (7) Å and a slippage of 1.163 Å.

## Database survey   

A search of the Cambridge Structural Database (Version 5.36; last update November 2014; Groom & Allen, 2014 ▸) gave 20 hits for Schiff bases derived from pyrene-1-carbaldehyde. A search for Schiff base compounds involving *N*-phenyl-*p*-phenyl­enedi­amine gave three hits. Of these three compounds, *N* ^1^-phenyl-*N* ^4^-(quinolin-2-yl­methyl­ene)benzene-1,4-di­amine {synonym: *N*-phenyl-4-[(quinolin-2-yl­methyl­ene)amino]aniline; WOJJIQ; Faizi *et al.*, 2014 ▸} is the most similar to the title compound. Here the dihedral angles between the central benzene ring and the terminal phenyl ring and the quinoline ring system (r.m.s. deviation = 0.027 Å) are 44.72 (7) and 9.02 (4)°, respectively. In the title compound, the dihedral angles between the central benzene ring and the terminal phenyl ring and the pyrenyl ring system (r.m.s. deviation = 0.027 Å) are 43.43 (9) and 29.33 (7)°, respectively.

## Synthesis and crystallization   

80 mg (0.435 mmol) of *N*-phenyl-*p*-phenyl­enedi­amine were dissolved in 10 ml of absolute ethanol. To this solution, 100 mg (0.435 mmol) of pyrene-1-carbaldehyde in 5 ml of absolute ethanol was added dropwise under stirring. The mixture was stirred for 10 min, two drops of glacial acetic acid were then added and the mixture was further refluxed for 2h. The resulting yellow precipitate was recovered by filtration, washed several times with small portions of ice-cold ethanol and then with diethyl ether to give 150 mg (87%) of the title compound. Yellow block-like crystals suitable for X-ray analysis were obtained within 3 days by slow evaporation of a solution in MeOH.

## Refinement   

Crystal data, data collection and structure refinement details are summarized in Table 2 ▸. The NH and C-bound H atoms were located from difference Fourier maps and freely refined.

## Supplementary Material

Crystal structure: contains datablock(s) global, I. DOI: 10.1107/S2056989015001814/su5072sup1.cif

Structure factors: contains datablock(s) I. DOI: 10.1107/S2056989015001814/su5072Isup2.hkl

Supporting information file. DOI: 10.1107/S2056989015001814/su5072Isup3.cml

CCDC reference: 1045835

Additional supporting information:  crystallographic information; 3D view; checkCIF report

## Figures and Tables

**Table 1:** NH and CH interactions (, ) *Cg*5 and *Cg*6 are the centroids of the C20C24/C29 and C24C29 rings, respectively, in the pyrenyl ring system.

*D*H*A*	*D*H	H*A*	*D* *A*	*D*H*A*
N1H1*A* *Cg*5^i^	0.89(2)	2.80(2)	3.6524(19)	163(2)
C6H6*Cg*6^i^	0.99(2)	2.76(2)	3.631(2)	147(1)

Symmetry code: (i) 
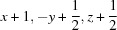
.

**Table 2:** Experimental details

Crystal data	
Chemical formula	C_29_H_20_N_2_
*M*_r_	396.47
Crystal system, space group	Monoclinic, *P*2_1_/*c*
Temperature (K)	100
*a*, *b*, *c* ()	13.0433(6), 12.2700(5), 13.4981(7)
()	114.269(2)
*V* (^3^)	1969.34(16)
*Z*	4
Radiation type	Mo *K*
(mm^1^)	0.08
Crystal size (mm)	0.18 0.14 0.12

Data collection	
Diffractometer	Bruker APEXII CCD
Absorption correction	Multi-scan (*SADABS*; Sheldrick, 2004 ▸)
*T*_min_, *T*_max_	0.986, 0.991
No. of measured, independent and observed [*I* > 2(*I*)] reflections	19561, 4882, 3015
*R*_int_	0.058
(sin /)_max_ (^1^)	0.668

Refinement	
*R*[*F* ^2^ > 2(*F* ^2^)], *wR*(*F* ^2^), *S*	0.056, 0.136, 1.02
No. of reflections	4882
No. of parameters	360
H-atom treatment	All H-atom parameters refined
_max_, _min_ (e ^3^)	0.27, 0.31

Computer programs: *SMART* and *SAINT* (Bruker, 2003 ▸), *SIR97* (Altomare *et al.*, 1999 ▸), *DIAMOND* (Brandenberg Putz, 2006 ▸), *Mercury* (Macrae *et al.*, 2008 ▸), *SHELXL97* (Sheldrick, 2015 ▸) and *PLATON* (Spek, 2009 ▸).

## supplementary crystallographic information

### Crystal data

**Table:**

C_29_H_20_N_2_	*F*(000) = 832
*M**_r_* = 396.47	*D*_x_ = 1.337 Mg m^−^^3^
Monoclinic, *P*2_1_/*c*	Mo *K*α radiation, λ = 0.71073 Å
Hall symbol: -P 2ybc	Cell parameters from 4765 reflections
*a* = 13.0433 (6) Å	θ = 2.5–28.1°
*b* = 12.2700 (5) Å	µ = 0.08 mm^−^^1^
*c* = 13.4981 (7) Å	*T* = 100 K
β = 114.269 (2)°	Block, yellow
*V* = 1969.34 (16) Å^3^	0.18 × 0.14 × 0.12 mm
*Z* = 4	

### Data collection

**Table:**

Bruker APEXII CCD diffractometer	4882 independent reflections
Radiation source: fine-focus sealed tube	3015 reflections with *I* > 2σ(*I*)
Graphite monochromator	*R*_int_ = 0.058
φ and ω scans	θ_max_ = 28.3°, θ_min_ = 2.4°
Absorption correction: multi-scan (*SADABS*; Sheldrick, 2004)	*h* = −13→17
*T*_min_ = 0.986, *T*_max_ = 0.991	*k* = −15→16
19561 measured reflections	*l* = −18→17

### Refinement

**Table:**

Refinement on *F*^2^	Primary atom site location: structure-invariant direct methods
Least-squares matrix: full	Secondary atom site location: difference Fourier map
*R*[*F*^2^ > 2σ(*F*^2^)] = 0.056	Hydrogen site location: inferred from neighbouring sites
*wR*(*F*^2^) = 0.136	All H-atom parameters refined
*S* = 1.02	*w* = 1/[σ^2^(*F*_o_^2^) + (0.0618*P*)^2^ + 0.3614*P*] where *P* = (*F*_o_^2^ + 2*F*_c_^2^)/3
4882 reflections	(Δ/σ)_max_ < 0.001
360 parameters	Δρ_max_ = 0.27 e Å^−^^3^
0 restraints	Δρ_min_ = −0.31 e Å^−^^3^

### Special details

**Table:**

Geometry. All e.s.d.'s (except the e.s.d. in the dihedral angle between two l.s. planes) are estimated using the full covariance matrix. The cell e.s.d.'s are taken into account individually in the estimation of e.s.d.'s in distances, angles and torsion angles; correlations between e.s.d.'s in cell parameters are only used when they are defined by crystal symmetry. An approximate (isotropic) treatment of cell e.s.d.'s is used for estimating e.s.d.'s involving l.s. planes.
Refinement. Refinement of *F*^2^ against ALL reflections. The weighted *R*-factor *wR* and goodness of fit *S* are based on *F*^2^, conventional *R*-factors *R* are based on *F*, with *F* set to zero for negative *F*^2^. The threshold expression of *F*^2^ > σ(*F*^2^) is used only for calculating *R*-factors(gt) *etc*. and is not relevant to the choice of reflections for refinement. *R*-factors based on *F*^2^ are statistically about twice as large as those based on *F*, and *R*- factors based on ALL data will be even larger.

### Fractional atomic coordinates and isotropic or equivalent isotropic displacement parameters (Å^2^)

**Table:**

	*x*	*y*	*z*	*U*_iso_*/*U*_eq_	
N2	0.81481 (12)	0.07101 (12)	0.38507 (11)	0.0234 (4)	
C27	0.53428 (14)	0.02897 (14)	0.16435 (13)	0.0185 (4)	
C17	0.49949 (14)	−0.16939 (14)	0.13951 (13)	0.0209 (4)	
C29	0.35640 (14)	−0.04116 (14)	0.02242 (13)	0.0185 (4)	
C14	0.64048 (14)	0.00667 (14)	0.24975 (13)	0.0192 (4)	
C20	0.28576 (14)	−0.13001 (14)	−0.03213 (13)	0.0209 (4)	
C15	0.67242 (15)	−0.10188 (15)	0.27839 (14)	0.0223 (4)	
C10	0.89081 (14)	0.15581 (14)	0.43730 (13)	0.0216 (4)	
C13	0.71978 (14)	0.09325 (15)	0.30778 (13)	0.0211 (4)	
N1	1.12616 (13)	0.39778 (14)	0.60829 (12)	0.0289 (4)	
C6	1.28287 (16)	0.47750 (15)	0.75618 (15)	0.0262 (4)	
C18	0.42695 (15)	−0.25720 (15)	0.08151 (15)	0.0237 (4)	
C28	0.46352 (13)	−0.06019 (14)	0.10921 (12)	0.0177 (4)	
C23	0.21349 (15)	0.08409 (15)	−0.09503 (13)	0.0223 (4)	
C25	0.39177 (15)	0.15502 (15)	0.04656 (13)	0.0207 (4)	
C24	0.31949 (14)	0.06726 (14)	−0.00974 (13)	0.0186 (4)	
C1	1.18326 (14)	0.41717 (14)	0.71999 (13)	0.0218 (4)	
C16	0.60457 (15)	−0.18739 (15)	0.22482 (14)	0.0227 (4)	
C21	0.18068 (15)	−0.10853 (15)	−0.11566 (14)	0.0227 (4)	
C19	0.32537 (15)	−0.23874 (15)	0.00075 (15)	0.0248 (4)	
C8	1.04477 (14)	0.21599 (15)	0.60259 (14)	0.0225 (4)	
C2	1.14291 (15)	0.38326 (15)	0.79601 (14)	0.0218 (4)	
C26	0.49341 (14)	0.13708 (15)	0.12909 (13)	0.0205 (4)	
C3	1.20224 (16)	0.40741 (15)	0.90521 (14)	0.0243 (4)	
C22	0.14500 (15)	−0.00301 (15)	−0.14681 (14)	0.0242 (4)	
C9	0.96797 (14)	0.13737 (16)	0.54378 (14)	0.0224 (4)	
C4	1.30180 (16)	0.46528 (15)	0.94087 (15)	0.0277 (4)	
C7	1.04841 (14)	0.31582 (15)	0.55524 (14)	0.0230 (4)	
C12	0.97321 (15)	0.33348 (16)	0.44705 (14)	0.0255 (4)	
C5	1.34123 (17)	0.50082 (16)	0.86574 (15)	0.0288 (5)	
C11	0.89664 (15)	0.25524 (16)	0.38926 (14)	0.0251 (4)	
H1A	1.1460 (18)	0.4403 (18)	0.5660 (18)	0.047 (7)*	
H11	0.8478 (13)	0.2685 (13)	0.3158 (14)	0.018 (4)*	
H15	0.7446 (15)	−0.1160 (14)	0.3384 (14)	0.023 (5)*	
H9	0.9661 (14)	0.0706 (15)	0.5772 (14)	0.021 (5)*	
H8	1.0955 (14)	0.2013 (14)	0.6739 (14)	0.021 (5)*	
H16	0.6282 (14)	−0.2600 (15)	0.2463 (14)	0.021 (5)*	
H6	1.3112 (15)	0.5027 (15)	0.7024 (15)	0.030 (5)*	
H2	1.0753 (15)	0.3456 (14)	0.7734 (13)	0.020 (5)*	
H12	0.9774 (15)	0.4012 (16)	0.4143 (14)	0.028 (5)*	
H3	1.1711 (16)	0.3855 (15)	0.9576 (16)	0.034 (5)*	
H26	0.5406 (14)	0.1991 (15)	0.1644 (14)	0.021 (5)*	
H22	0.0715 (15)	0.0126 (14)	−0.2071 (14)	0.020 (5)*	
H18	0.4536 (14)	−0.3307 (15)	0.1048 (13)	0.022 (5)*	
H4	1.3450 (15)	0.4810 (14)	1.0175 (15)	0.029 (5)*	
H23	0.1887 (15)	0.1603 (15)	−0.1163 (14)	0.026 (5)*	
H5	1.4111 (17)	0.5434 (16)	0.8905 (16)	0.041 (6)*	
H21	0.1325 (14)	−0.1709 (14)	−0.1498 (13)	0.018 (4)*	
H19	0.2740 (15)	−0.2977 (15)	−0.0384 (15)	0.030 (5)*	
H13	0.6979 (14)	0.1714 (15)	0.2880 (14)	0.024 (5)*	
H25	0.3679 (14)	0.2288 (15)	0.0244 (14)	0.022 (5)*	

### Atomic displacement parameters (Å^2^)

**Table:**

	*U*^11^	*U*^22^	*U*^33^	*U*^12^	*U*^13^	*U*^23^
N2	0.0218 (8)	0.0270 (9)	0.0196 (7)	−0.0022 (7)	0.0067 (6)	−0.0027 (6)
C27	0.0209 (9)	0.0220 (10)	0.0159 (8)	0.0004 (7)	0.0110 (7)	−0.0008 (7)
C17	0.0223 (9)	0.0228 (10)	0.0225 (9)	0.0006 (8)	0.0142 (8)	0.0022 (7)
C29	0.0200 (9)	0.0212 (10)	0.0184 (8)	−0.0007 (7)	0.0119 (7)	−0.0010 (7)
C14	0.0199 (9)	0.0241 (10)	0.0161 (8)	0.0012 (7)	0.0099 (7)	−0.0009 (7)
C20	0.0212 (9)	0.0233 (10)	0.0222 (9)	−0.0035 (8)	0.0131 (7)	−0.0016 (7)
C15	0.0207 (9)	0.0289 (11)	0.0177 (9)	0.0035 (8)	0.0084 (7)	0.0027 (8)
C10	0.0176 (9)	0.0247 (10)	0.0227 (9)	0.0010 (8)	0.0087 (7)	−0.0021 (7)
C13	0.0223 (9)	0.0237 (10)	0.0191 (8)	0.0021 (8)	0.0103 (7)	−0.0016 (7)
N1	0.0320 (9)	0.0360 (10)	0.0192 (8)	−0.0125 (8)	0.0109 (7)	−0.0018 (7)
C6	0.0296 (10)	0.0274 (11)	0.0268 (9)	−0.0048 (9)	0.0170 (8)	−0.0038 (8)
C18	0.0284 (10)	0.0182 (10)	0.0294 (9)	−0.0011 (8)	0.0168 (8)	0.0022 (8)
C28	0.0198 (9)	0.0203 (10)	0.0164 (8)	0.0002 (7)	0.0111 (7)	0.0004 (7)
C23	0.0236 (9)	0.0247 (10)	0.0194 (9)	0.0042 (8)	0.0095 (7)	0.0025 (8)
C25	0.0252 (9)	0.0185 (10)	0.0205 (9)	0.0038 (8)	0.0115 (8)	0.0006 (7)
C24	0.0201 (9)	0.0223 (10)	0.0166 (8)	−0.0005 (8)	0.0108 (7)	−0.0016 (7)
C1	0.0224 (9)	0.0218 (10)	0.0206 (8)	0.0015 (8)	0.0081 (7)	−0.0006 (7)
C16	0.0259 (10)	0.0198 (10)	0.0242 (9)	0.0032 (8)	0.0122 (8)	0.0055 (8)
C21	0.0210 (9)	0.0273 (11)	0.0221 (9)	−0.0059 (8)	0.0112 (8)	−0.0052 (8)
C19	0.0267 (10)	0.0217 (10)	0.0304 (10)	−0.0070 (9)	0.0163 (8)	−0.0041 (8)
C8	0.0186 (9)	0.0290 (11)	0.0181 (9)	0.0043 (8)	0.0057 (7)	−0.0007 (8)
C2	0.0192 (9)	0.0209 (10)	0.0243 (9)	0.0017 (8)	0.0080 (8)	0.0002 (7)
C26	0.0235 (9)	0.0191 (10)	0.0199 (9)	−0.0018 (8)	0.0100 (8)	−0.0027 (7)
C3	0.0295 (10)	0.0214 (10)	0.0240 (9)	0.0037 (8)	0.0131 (8)	0.0007 (8)
C22	0.0194 (9)	0.0331 (11)	0.0190 (9)	−0.0004 (8)	0.0069 (7)	−0.0008 (8)
C9	0.0216 (9)	0.0223 (10)	0.0228 (9)	0.0027 (8)	0.0086 (8)	0.0005 (8)
C4	0.0317 (11)	0.0263 (11)	0.0201 (9)	0.0004 (9)	0.0057 (8)	−0.0055 (8)
C7	0.0214 (9)	0.0294 (11)	0.0206 (8)	−0.0029 (8)	0.0109 (7)	−0.0051 (8)
C12	0.0264 (10)	0.0286 (11)	0.0227 (9)	−0.0009 (9)	0.0113 (8)	0.0024 (8)
C5	0.0262 (10)	0.0277 (11)	0.0306 (10)	−0.0054 (9)	0.0097 (9)	−0.0091 (8)
C11	0.0212 (9)	0.0345 (11)	0.0169 (8)	−0.0004 (9)	0.0052 (7)	−0.0002 (8)

### Geometric parameters (Å, º)

**Table:**

N2—C13	1.279 (2)	C23—C22	1.384 (3)
N2—C10	1.410 (2)	C23—C24	1.404 (2)
C27—C14	1.418 (2)	C23—H23	0.992 (18)
C27—C28	1.428 (2)	C25—C26	1.354 (2)
C27—C26	1.436 (2)	C25—C24	1.427 (2)
C17—C16	1.399 (2)	C25—H25	0.964 (18)
C17—C28	1.423 (2)	C1—C2	1.395 (2)
C17—C18	1.435 (2)	C16—H16	0.949 (18)
C29—C24	1.421 (2)	C21—C22	1.382 (3)
C29—C20	1.422 (2)	C21—H21	0.977 (17)
C29—C28	1.426 (2)	C19—H19	0.980 (19)
C14—C15	1.402 (2)	C8—C9	1.382 (2)
C14—C13	1.465 (2)	C8—C7	1.391 (3)
C20—C21	1.396 (2)	C8—H8	0.934 (17)
C20—C19	1.434 (3)	C2—C3	1.386 (2)
C15—C16	1.371 (3)	C2—H2	0.928 (18)
C15—H15	0.972 (17)	C26—H26	0.972 (18)
C10—C9	1.393 (2)	C3—C4	1.381 (3)
C10—C11	1.398 (3)	C3—H3	0.99 (2)
C13—H13	1.004 (18)	C22—H22	0.988 (17)
N1—C7	1.398 (2)	C9—H9	0.941 (18)
N1—C1	1.401 (2)	C4—C5	1.383 (3)
N1—H1A	0.89 (2)	C4—H4	0.972 (18)
C6—C5	1.387 (3)	C7—C12	1.401 (2)
C6—C1	1.397 (2)	C12—C11	1.374 (3)
C6—H6	0.990 (19)	C12—H12	0.954 (19)
C18—C19	1.344 (2)	C5—H5	0.98 (2)
C18—H18	0.971 (18)	C11—H11	0.947 (16)
			
C13—N2—C10	119.79 (15)	C2—C1—C6	118.58 (16)
C14—C27—C28	118.84 (15)	C2—C1—N1	123.14 (16)
C14—C27—C26	123.60 (16)	C6—C1—N1	118.22 (16)
C28—C27—C26	117.57 (15)	C15—C16—C17	120.99 (17)
C16—C17—C28	118.76 (16)	C15—C16—H16	120.0 (10)
C16—C17—C18	122.26 (16)	C17—C16—H16	119.0 (10)
C28—C17—C18	118.98 (15)	C22—C21—C20	121.25 (17)
C24—C29—C20	119.58 (15)	C22—C21—H21	121.3 (9)
C24—C29—C28	119.93 (15)	C20—C21—H21	117.5 (10)
C20—C29—C28	120.49 (16)	C18—C19—C20	121.16 (17)
C15—C14—C27	119.20 (16)	C18—C19—H19	122.7 (11)
C15—C14—C13	118.50 (15)	C20—C19—H19	116.2 (11)
C27—C14—C13	122.28 (16)	C9—C8—C7	120.27 (16)
C21—C20—C29	119.05 (16)	C9—C8—H8	119.8 (11)
C21—C20—C19	122.34 (16)	C7—C8—H8	120.0 (11)
C29—C20—C19	118.61 (16)	C3—C2—C1	120.25 (18)
C16—C15—C14	121.83 (16)	C3—C2—H2	119.7 (11)
C16—C15—H15	119.8 (10)	C1—C2—H2	120.0 (11)
C14—C15—H15	118.4 (10)	C25—C26—C27	121.84 (17)
C9—C10—C11	117.78 (16)	C25—C26—H26	119.0 (10)
C9—C10—N2	117.22 (16)	C27—C26—H26	119.1 (10)
C11—C10—N2	124.96 (15)	C4—C3—C2	120.95 (18)
N2—C13—C14	121.06 (17)	C4—C3—H3	119.7 (11)
N2—C13—H13	119.6 (10)	C2—C3—H3	119.3 (11)
C14—C13—H13	119.3 (10)	C21—C22—C23	120.21 (16)
C7—N1—C1	128.77 (17)	C21—C22—H22	121.6 (10)
C7—N1—H1A	115.8 (14)	C23—C22—H22	118.2 (10)
C1—N1—H1A	115.2 (14)	C8—C9—C10	121.67 (18)
C5—C6—C1	120.40 (18)	C8—C9—H9	119.2 (10)
C5—C6—H6	120.7 (11)	C10—C9—H9	119.1 (11)
C1—C6—H6	118.9 (11)	C3—C4—C5	119.10 (17)
C19—C18—C17	121.66 (18)	C3—C4—H4	121.5 (11)
C19—C18—H18	121.4 (10)	C5—C4—H4	119.4 (11)
C17—C18—H18	116.9 (10)	C8—C7—N1	123.53 (16)
C17—C28—C27	120.37 (15)	C8—C7—C12	118.25 (16)
C17—C28—C29	119.10 (15)	N1—C7—C12	118.16 (17)
C27—C28—C29	120.53 (15)	C11—C12—C7	121.18 (18)
C22—C23—C24	120.94 (17)	C11—C12—H12	120.8 (11)
C22—C23—H23	121.0 (10)	C7—C12—H12	118.0 (11)
C24—C23—H23	118.0 (10)	C4—C5—C6	120.69 (18)
C26—C25—C24	121.64 (17)	C4—C5—H5	119.4 (12)
C26—C25—H25	119.3 (10)	C6—C5—H5	119.9 (12)
C24—C25—H25	119.0 (10)	C12—C11—C10	120.79 (16)
C23—C24—C29	118.96 (16)	C12—C11—H11	119.6 (10)
C23—C24—C25	122.55 (16)	C10—C11—H11	119.6 (10)
C29—C24—C25	118.50 (15)		
			
C28—C27—C14—C15	−0.7 (2)	C26—C25—C24—C29	−0.1 (2)
C26—C27—C14—C15	179.71 (15)	C5—C6—C1—C2	1.4 (3)
C28—C27—C14—C13	177.73 (15)	C5—C6—C1—N1	178.65 (17)
C26—C27—C14—C13	−1.9 (3)	C7—N1—C1—C2	−24.6 (3)
C24—C29—C20—C21	−0.7 (2)	C7—N1—C1—C6	158.27 (18)
C28—C29—C20—C21	179.03 (15)	C14—C15—C16—C17	−0.7 (3)
C24—C29—C20—C19	179.20 (15)	C28—C17—C16—C15	−0.3 (3)
C28—C29—C20—C19	−1.1 (2)	C18—C17—C16—C15	179.62 (17)
C27—C14—C15—C16	1.2 (3)	C29—C20—C21—C22	0.8 (3)
C13—C14—C15—C16	−177.34 (16)	C19—C20—C21—C22	−179.15 (16)
C13—N2—C10—C9	154.37 (16)	C17—C18—C19—C20	1.0 (3)
C13—N2—C10—C11	−27.7 (3)	C21—C20—C19—C18	−179.97 (17)
C10—N2—C13—C14	178.13 (15)	C29—C20—C19—C18	0.1 (3)
C15—C14—C13—N2	−2.0 (2)	C6—C1—C2—C3	−1.3 (3)
C27—C14—C13—N2	179.52 (16)	N1—C1—C2—C3	−178.42 (17)
C16—C17—C18—C19	178.95 (17)	C24—C25—C26—C27	0.2 (3)
C28—C17—C18—C19	−1.2 (3)	C14—C27—C26—C25	179.47 (16)
C16—C17—C28—C27	0.7 (2)	C28—C27—C26—C25	−0.1 (2)
C18—C17—C28—C27	−179.21 (15)	C1—C2—C3—C4	0.1 (3)
C16—C17—C28—C29	−179.90 (15)	C20—C21—C22—C23	−0.1 (3)
C18—C17—C28—C29	0.2 (2)	C24—C23—C22—C21	−0.6 (3)
C14—C27—C28—C17	−0.2 (2)	C7—C8—C9—C10	−1.4 (3)
C26—C27—C28—C17	179.41 (15)	C11—C10—C9—C8	2.9 (3)
C14—C27—C28—C29	−179.59 (15)	N2—C10—C9—C8	−178.95 (15)
C26—C27—C28—C29	0.0 (2)	C2—C3—C4—C5	1.1 (3)
C24—C29—C28—C17	−179.38 (15)	C9—C8—C7—N1	−177.74 (17)
C20—C29—C28—C17	0.9 (2)	C9—C8—C7—C12	−0.5 (3)
C24—C29—C28—C27	0.0 (2)	C1—N1—C7—C8	−25.3 (3)
C20—C29—C28—C27	−179.71 (15)	C1—N1—C7—C12	157.42 (18)
C22—C23—C24—C29	0.6 (2)	C8—C7—C12—C11	0.9 (3)
C22—C23—C24—C25	−179.12 (16)	N1—C7—C12—C11	178.29 (17)
C20—C29—C24—C23	0.1 (2)	C3—C4—C5—C6	−1.0 (3)
C28—C29—C24—C23	−179.69 (15)	C1—C6—C5—C4	−0.3 (3)
C20—C29—C24—C25	179.77 (15)	C7—C12—C11—C10	0.6 (3)
C28—C29—C24—C25	0.0 (2)	C9—C10—C11—C12	−2.5 (3)
C26—C25—C24—C23	179.57 (16)	N2—C10—C11—C12	179.53 (17)

### Hydrogen-bond geometry (Å, º)

Cg5 and Cg6 are the centroids of the C20–C24/C29 and C24–C29 rings, respectively, in the pyrenyl ring system.

**Table:**

*D*—H···*A*	*D*—H	H···*A*	*D*···*A*	*D*—H···*A*
N1—H1*A*···*Cg*5^i^	0.89 (2)	2.80 (2)	3.6524 (19)	163 (2)
C6—H6···*Cg*6^i^	0.99 (2)	2.76 (2)	3.631 (2)	147 (1)

Symmetry code: (i) *x*+1, −*y*+1/2, *z*+1/2.
